# Wiring of cellular proteostasis by J-domain proteins

**DOI:** 10.1016/j.cstres.2026.100191

**Published:** 2026-05-22

**Authors:** Matthias P. Mayer

**Affiliations:** Center for Molecular Biology of Heidelberg University (ZMBH), DKFZ-ZMBH Alliance, Heidelberg, Germany

**Keywords:** Chaperones, Hsp70, Protein folding, Amyloid fibrils, Neurodegenerative diseases

## Abstract

Originally J-domain proteins (JDPs) were viewed as accessory co-chaperones of 70 kDa heat shock proteins (Hsp70s), the actual chaperones, stimulating ATPase activity of Hsp70s when a protein substrate is bound. This view apparently underestimates the role of JDPs, as most of the decisions within the Hsp70 network seems to be taken at the level of JDPs. The JDPs are the brain, so to speak, and the Hsp70s are the muscles of this chaperone network.

## Introduction: JDPs and the Hsp70 chaperone machinery

**Fig. 1:**
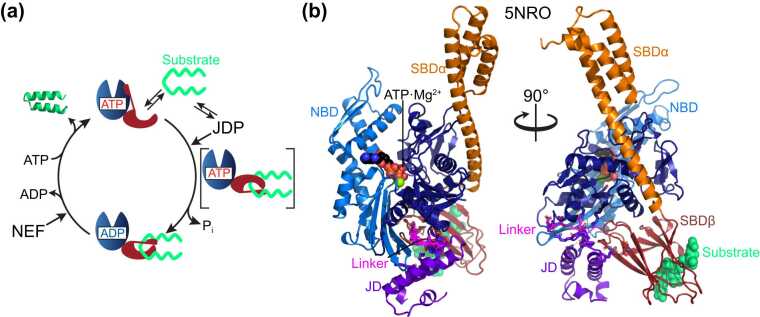
*JDP-Hsp70 interaction*. (a) Hsp70 chaperone cycle. The substrate protein binds with high association rates to the ATP-bound state of Hsp70, in which the substrate binding groove is freely accessible and both subdomains of the substrate binding domain (SBD, dark red) are docked onto the nucleotide binding domain (NBD, blue). The affinity of the ATP-bound state of Hsp70 to substrates is too low to prevent aggregation of misfolded proteins. However, substrate and a JDP synergistically stimulate Hsp70’s ATPase activity, leading to the closure of the lid (SBDα) and the trapping of the substrate and, consequently, transition to the high-affinity state of Hsp70. A crystal structure suggests a pre-hydrolysis conformation that may or may not be on path to the high-affinity state (in square brackets).^12^ After ATP hydrolysis, nucleotide exchange becomes rate limiting for substrate release. Nucleotide exchange factors (NEF) catalyze the release of ADP and allow rebinding of ATP, leading to reopening of the substrate binding pocket and release of the substrate. Thus, NEFs regulate the lifetime of the Hsp70-substrate complex. (b) Cartoon representation of the crystal structure of the *E coli* Hsp70 DnaK in complex with the J-domain of *E coli* DnaJ (5NRO^13^), overlayed with the SBDβ of the crystal structure of the SBD of DnaK in complex with a substrate peptide (only peptide shown; 1DKX^14^). The two lobes of the NBD are colored in dark and light blue; the linker between NBD and SBD that is important for allostery and interaction with the J-domain in magenta; SBDβ, dark red; SBDα, orange; J-domain, purple; substrate peptide as spheres in green; ATP as spheres in atom colors; residues important for the interaction between Hsp70 and the J-domain and for allosteric signal transmission to the catalytic center as sticks in atom colors with carbon in the color of the corresponding domain.

The Hsp70 chaperone machineries consist of Hsp70s, JDPs (formerly also called J-proteins, DnaJ proteins, or Hsp40s), and nucleotide exchange factors (Figure 1a). They constitute central hubs in the cellular protein quality surveillance network, controlling a wide variety and diversity of protein folding processes, from *de novo* folding of newly synthesized polypeptides to refolding of misfolded or denatured proteins; to disassembly of native protein complexes (e.g., clathrin cages or trimeric HSF1), amorphous aggregates, and amyloid fibrils; and to degradation of proteins (for review see^1^). Hsp70 chaperones could be called, in a wider sense, protein conformation isomerases: they destabilize some conformations (also called “unfoldase” activity) as shown by hydrogen-exchange mass spectrometry and protease protection experiments^2^, ^3^ and stabilize other conformations as shown by optical tweezer experiments.^4^ Although some differences have been observed between Hsp70s,^5^, ^6^, ^7^ the real diversity resides within the plethora of JDPs. In fact, the JDPs represent by far the largest family of molecular chaperones, with currently more than 200,000 sequences in databases.^8^ The number of JDP encoding genes in genomes has significantly expanded from prokaryotes to multicellular eukaryotes. Whereas *Escherichia coli* has three *bona fide* Hsp70s and six JDPs, and 90% of 1709 bacterial genomes contain 6 or less JDP sequences,^9^ *Saccharomyces cerevisiae* has 10 Hsp70s and 22 JDPs, *Drosophila melanogaster* has 12 Hsp70s and 21 JDPs, and humans have 7 *bona fide* Hsp70s and 49 JDPs (not counting splice variants).^10^ Of note, *Plasmodium falciparum* produces four Hsp70s and 49 JDPs, of which 17 are predicted to be exported into the host erythrocyte,^11^ suggesting that not only a complex cellular organization but also a complex life cycle may favor the evolution of a large number and variety of JDPs.

**Fig. 2:**
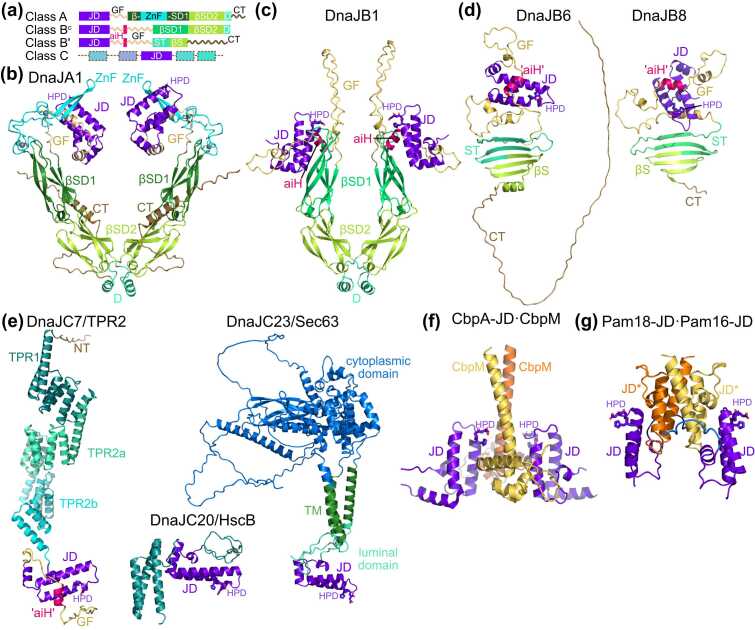
*Overview of JDP structures*. (a) Domain organization of class A, canonical class B (class B^c^), non-canonical class B (class B’), and class C JDPs; size of the domain boxes and disordered regions (wavy lines) approximately at scale; J-domain (JD), purple; glycine-phenylalanine rich region (GF), light orange; zinc-finger-like domain (ZnF), cyan; β-sandwich domains (βSD1 and βSD2), shades of green; dimerization domain (D), greencyan; disordered C-terminal tail (CT), brown; autoinhibitory helix (aiH, often called helix 5), pink; serine-threonine-rich region (ST), lime green; β-sheet domain (βS), yellowgreen. Whereas class A and class B JDPs have the J-domain close to the N-terminus, class C JDPs may have their J-domain anywhere within the sequence and contain often a number of additional domains not related to the domains of class A and class B JDPs N- or C-terminal or on both sides of their J-domain (illustrated as dashed differently colored boxes). (b) Example of a class A JDP. Cartoon representation of an AlphaFold 3^19^ model of human DnaJA1 colored according to the color scheme in A; position of the J-domain is arbitrary as it is tethered to the remainder of the protein by the long flexible GF-region (ca. 150 Å if extended); the HPD motif shown as sticks in atom colors with carbon in purple; Zn^2+^ shown as spheres in gray, Zn^2+^-coordinating cysteines shown as sticks. (c) Example of a canonical class B JDP. Cartoon representation of an AlphaFold 3^19^ model of human DnaJB1 colored according to the color scheme in A. (d) Examples of non-canonical class B’ JDPs. Cartoon representation of AlphaFold 3^19^ models of human DnaJB6 and DnaJB8 colored according to the color scheme in A; ‘aiH’, putative autoinhibitory helix. (e) Examples of class C JDPs. Cartoon representation of AlphaFold 3^19^ models of human cytosolic DnaJC7/TPR2, mitochondrial matrix DnaJC20/HscB, and endoplasmic reticulum DnaJC23/Sec63; NT, N-terminal tail; TPR, tetratricopeptide repeat domains, different shades of greencyan/teal; J-domains (JD), putative autoinhibitory helix (‘aiH’) and GF-region colored as in A; TM, transmembrane helices, dark green. (f) Cartoon representation of the crystal structure of the complex of two copies of the J-domain (purple) of the *E coli* class B JDP CbpA with a dimer of its inhibitor CbpM (orange and light orange). Indicated are the HPD-motifs as sticks facing CbpM. (g) Cartoon representation of the crystal structure of the complex of the J-domain (purple) of the mitochondrial pre-sequence import motor-associated JDP Pam18 and the pseudo-J-domain (orange and light orange) of its interaction partner Pam16 in a dimer of dimers assembly.

JDPs are modular multidomain proteins that are characterized by the J-domain, a 60–70 residue α-helical hairpin, with the signature histidine-proline-aspartate (HPD) motif within the apical loop of the hairpin. The J-domain with the HPD motif is essential for stimulating the ATPase activity of Hsp70s, and even conservative amino acid replacements like D→N or H→Q within this motif abrogate the ability to stimulate Hsp70’s ATPase activity and chaperone function^15^, ^16^ (Figure 1b). As an operational classification, JDPs were divided into three types or classes according to the domains they share with the prototypic JDP, *E coli* DnaJ (Figure 2a).^17^ In type I/class A JDPs the N-terminal J-domain is followed by an unstructured glycine-phenylalanine rich region (G/F) of 30–40 amino acids, two homologous twisted β-sandwich domains, βSD1 and βSD2, with a zinc-finger like β-hairpin (ZnF) inserted into βSD1, a helical dimerization domain, and a C-terminal unstructured tail of some 30 amino acids in prokaryotes and 50–100 amino acids in eukaryotes (Figure 2b). Type II/class B JDPs were originally defined as having an N-terminal J-domain and a generally longer (70–100 amino acids) G/F-rich region but no ZnF.^17^ A subset of class B JDPs, the so-called canonical class B JDPs, have C-terminal to the G/F region two β-sandwich domains, βSD1 and βSD2, and a C-terminal dimerization domain that are structurally very similar to the βSD and dimerization domain of class A JDPs (Figure 2c). The non-canonical class B JDPs have unrelated domains C-terminal to the G/F-rich region (Figure 2d). Type III/class C JDPs only share the J-domain, and this domain is not necessarily at the N-terminus as in class A and class B JDPs but may be anywhere within the sequence (Figure 2a & e). Whereas the length of class A and class B JDPs is rather homogeneous with some 380 (class A) and 250–350 (class B) residues, the length of class C JDPs varies between 54 and more than 12,000 residues according to sequencing data. More recently, a fourth type of JDPs has been defined as containing a clearly recognizable J-domain but in which the HPD motif is non-conservatively replaced.^18^ These proteins therefore may not promote Hsp70s’ protein folding functions or not cooperate with Hsp70s at all. There are many of these pseudo-JDPs that would otherwise be classified into class C JDPs (e.g., Pam16) but also at least one from the domain composition class B JDP (DnaJB13). The functional relevance of this type of pseudo-JDPs in the context of the Hsp70 network is not yet elucidated.

Although this classification is practical, it does neither reflect an evolutionary nor a functional relationship. Apparently, eukaryotic cells only inherited class A JDPs from the last common ancestor of prokaryotes and eukaryotes and class B JDPs evolved several times independently from class A JDPs.^20^ Class A and class B JDPs were considered generalists that interact with a large variety of non-native and aggregated proteins and with native proteins that have non-native like features, whereas class C JDPs were considered specialists that either interact with one or a small number of similar proteins or bind to locations like the ribosome and translocation pores where Hsp70 substrates emerge. Recent research draws a much more differentiated picture of the substrate recognition process as discussed below in the section on substrate recognition. There is also evidence that some JDPs have a chaperone function of their own, and the interaction with Hsp70s is not essential and more like an added bonus (see section on substrate recognition). Moreover, a recent publication suggests that in some cases the interaction of the JDP-bound substrate with Hsp70s is bypassed with the aid of a special cochaperone (see the below section on substrate processing by JDPs bypassing Hsp70s). All of these observations indicate that it is time to revise our view on JDPs.

## JDP – Hsp70 interaction

Since the crystal structure of the J-domain of *E coli* DnaJ in complex with the *E coli* Hsp70 DnaK in the ATP-bound state was solved, the functional interaction of JDPs with Hsp70s seems to be elucidated^13^ (Figure 1b). In this structure, DnaK is in the domain-docked conformation, with the substrate binding domain (SBD) separated into the β-sandwich subdomain (SBDβ), wide open substrate binding groove, and the α-helical lid subdomain (SBDα), and both subdomains docked onto different faces of the nucleotide binding domain (NBD). The J-domain is docked onto the NBD-SBDβ-interface interacting with residues in both domains and in particular, the highly conserved linker between NBD and SBD, contacting a hydrogen network that converges on the γ-phosphate of ATP in the catalytic center. However, more recent proximity fluorescence quenching data demonstrate that JDPs do more than only stimulate the ATPase activity of Hsp70s. They facilitate substrate binding and affect the conformation of the Hsp70-substrate complex, preventing the substrate binding lid from closure. A functional J-domain is essential for this effect.^21^ The principle of J-domain-Hsp70 interaction is conserved, as suggested by multiple sequence alignment of J-domains and as many J-domains of diverse organisms and viruses have been grafted onto DnaJ replacing the endogenous J-domain, and shown to functionally interact with DnaK in complementation assays.^22^, ^23^ But, there are also structural and functional differences, as not all DnaK-interacting residues in DnaJ are conserved throughout the evolution of JDPs and as there are specific pairs of JDPs and Hsp70s that appear to have exclusive interactions (e.g., *S cerevisiae* Scj1-Kar2^24^; *E coli* HscB-HscA^25^; *E coli* DjlB/C-HscC^26^; *S cerevisiae* Hsc20-Ssq1^27^). Furthermore, there is evidence that even closely related class A and class B JDPs, like DnaJA1 and DnaJA2 or DnaJB1 and DnaJB4, cooperate with human Hsp70 in refolding of a denatured model substrate with very different efficiencies.^28^ Whether such differences are due to the specific J-domain-Hsp70 interaction is not clear. Similarly, yeast class A Ydj1 and class B Sis1 target the yeast Hsp70 Ssa1 with different efficacies to protein aggregates.^29^ However, these differences are more likely due to a different interaction mode that was more recently discovered.

Eukaryotic canonical class B JDPs contain an autoinhibitory helix within the G/F region that binds across the J-domain preventing interaction with Hsp70s.^30^ Interaction of the C-terminal EEVD motif, which is highly conserved in eukaryotic cytosolic Hsp70s, with the βSD1 of the JDP is necessary to unlock the JDP to allow functional interaction and stimulation of Hsp70’s ATPase activity. Deletion of the EEVD motif in yeast and human Hsp70s prevents refolding of misfolded proteins in cooperation with class B JDPs but not in cooperation with class A JDPs.^31^, ^32^ Mutation of the inhibitory helix partially circumvents this inhibition with respect to chaperoning of misfolded proteins but not for disassembling amyloid fibrils.^30^ It is currently not clear why this locking-unlocking mechanism of the J-domain of class B JDPs is necessary for amyloid fiber disassembly and whether this is also important for other functions of Hsp70s.

A recent NMR study on the class B DnaJ of *Thermus thermophilus* found that the segments within the G/F-rich region adjacent to the sequence that encompasses a similar autoinhibitory helix was also able to bind in a substrate-like fashion into the substrate binding pocket of *T thermophilus* DnaK’s SBDβ and, alternatively, with misfolded substrates when the autoinhibitory helix was released by single amino acid replacements, but not in the wildtype protein.^33^ Although prokaryotic Hsp70s do not end in an EEVD motif, their C-terminal intrinsically disordered tail was able to interact with the substrate binding site of *T thermophilus* DnaJ, albeit βSD2 not βSD1 as in the case of human DnaJB1, and to release the autoinhibition on the J-domain, freeing the autoinhibitory segment of the G/F-rich region. Thus, releasing the autoinhibitory helix by interaction of the C-terminal tail of Hsp70 with the substrate binding sites in the class B JDP could release autoinhibition and provide additional interaction sites for binding to the substrate.^33^

Inhibition of the J-domain of a JDP by a regulator was also found in prokaryotic JDPs. The J-domain of the canonical class B JDP CbpA (curved DNA-binding protein A) of *E coli* interacts with the small protein CbpM, which is encoded with CbpA in an operon and expressed like CbpA mostly in late stationary phase.^34^ This interaction prevents CbpA’s cochaperone function, as CbpM’s binding interface with CbpA’s J-domain overlaps with the interaction interface of the J-domain with DnaK^35^, ^36^ (Figure 2f).

A different type of regulation appears to be operative at the mitochondrial import motor for the Tim23 channel, guiding proteins into the matrix of mitochondria. The fully functional J-domain of the class C JDP Pam18 forms a stable complex with the J-domain of Pam16, which harbors mutations in the HPD motif that renders it unable to stimulate the ATPase activity of Hsp70^37^(Figure 2g). Interestingly, both JDPs are essential in yeast,^38^, ^39^ however, a combination of genetic and biochemical experiments led to the conclusion that an inhibition of Pam18 by Pam16 is not critical for yeast cells.^40^, ^41^, ^42^ Whether the Pam16-Pam18 interaction only serves to locate Pam18 close to the TIM17/TIM23 translocon appears to be unlikely, as such a purpose could have been achieved by much simpler means, for example, by direct interaction of the transmembrane domain of Pam18 with the translocon, unless Pam18 engages in additional, translocon-independent functions for which it acts independently of Pam16. It is also interesting that the Hsp70 interacting face of Pam18’s J-domain is not obstructed by interaction with the pseudo-J-domain of Pam16. The molecular mechanism of this J-domain tandem remains enigmatic so far.

Eukaryotic class A and class B JDPs are able to assemble into complexes by interaction of the J-domain of one JDP with the βSD1 of the second JDP. These complexes potentiate the activity of Hsp70s in solubilization of amorphous protein aggregates but not in disassembly of amyloid fibrils.^43^, ^44^ By themselves class A and class B, JDPs target Hsp70 to different types of aggregates. For example, human Hsc70 in cooperation with DnaJA2 preferentially solubilizes smaller luciferase aggregates of approximately 200–700 kDa, corresponding to some 3–10 luciferase molecules, whereas Hsc70 in cooperation with DnaJB1 preferentially solubilizes larger luciferase aggregates of approximately 700 to ≥5000 kDa, corresponding to some 10 to ≥ 80 luciferase molecules, assuming spherical assemblies.^45^ However, efficient disaggregation requires the cooperation between the two JDP classes. These cooperations seem to be very specific and could also be observed *in vivo* and in cell culture by proximity ligation and genetics.^43^, ^46^

## Substrate recognition and JDP mode of action

One hallmark of the Hsp70 machinery is the ability to interact with practically all proteins in their unfolded state, during *de novo* synthesis at the ribosome or during translocation through biological membranes, and with many proteins in their denatured state, but only with an exclusive set of native proteins. The elucidation of the recognition motif of *E coli* DnaK seemed to have solved this conundrum, as DnaK and presumably many Hsp70s prefer segments of up to five hydrophobic amino acids flanked by positively charged residues, a degenerative motif found in the hydrophobic core of most well-folded proteins and exposed only during *de novo* synthesis and in the denatured state.^47^ Such a scenario suggests that the Hsp70 selects the binding site and thus discriminates between denatured and native proteins. However, this binding preference was determined in the ADP-bound high-affinity state of DnaK that has very low substrate association rates (complex formation half-life of hours). To be able to compete with side reactions of protein folding such as aggregation, Hsp70s must bind to their substrates with the high association rates of their ATP-bound, low-affinity state and concomitantly hydrolyze ATP to transit to the high-affinity state, thereby trapping the substrate (Figure 1a). This mode of binding that is rapid enough to compete with aggregation and that increases apparent affinity by up to 3 orders of magnitude was coined ultra-affinity.^48^ The necessary high ATP hydrolysis rates are only achieved if Hsp70 interacts simultaneously with the protein substrate and a JDP. A simple solution for this problem is that the JDP binds to the substrate itself or is localized in close proximity to the substrate. This transfers the act of substrate selection to the JDP. Such a task seems to be relatively straightforward for highly specific class C JDPs like DnaJC20/Hsc20/Jac1, which evolved a specialized binding interface for the single substrate IscU/Isu1, recruiting Hsp70 for the process of Fe-S-cluster transfer to oxidoreductases,^49^, ^50^ or DnaJC6/auxilin, which only binds to clathrin cages and is essential for Hsc70-mediated uncoating of clathrin-coated endocytic vesicles.^51^, ^52^ However, the recognition of misfolded and aggregated proteins or amyloid fibrils and the distinction to native proteins seems to be more challenging as individual substrates in an ensemble of misfolded conformations are expected to expose distinct substrate and conformation-specific features.

For substrate recognition class A and canonical class B JDPs have multiple low-affinity binding sites with different properties. The peptide binding groove in βSD1 seems to prefer large hydrophobic and aromatic amino acid side chains consistent with early peptide library scanning experiments,^53^ but hydrogen bonding was also revealed in an NMR study.^54^ This also applies to the binding groove in βSD2 of canonical class B JDPs but not for class A JDPs, as this site appears to be occupied by the long C-terminal tail of these JDPs (Figure 2b), the function of which is currently unknown.^55^ Thus, dimeric canonical class B JDPs with two βSDs per protomer would have four low-affinity substrate binding grooves. In addition to these substrate binding sites, the hinge region between βSD1 and βSD2, including a crucial histidine within this region of DnaJB1 was demonstrated to be the binding site for the proline-rich domain that is C-terminally adjacent to the polyglutamine stretch of the Corea Huntington-causing polyQ protein fragment huntingtin exon 1 and essential for prevention of aggregation and disaggregation by Hsp70s.^56^ Interestingly, amino acid replacement of the histidine incapacitated the ability of DnaJB1 to prevent—in conjunction with Hsp70—aggregation of huntingtin exon 1-Q_48_ but not refolding of denatured luciferase or aggregation of the Alzheimer’s disease hallmark Aβ_42_, indicating that DnaJB1 relies on distinct binding sites for different substrates. Furthermore, as mentioned above, a recent study provided evidence that segments within the G/F-rich region could also interact with misfolded client proteins, adding highly flexible low-affinity interaction sites to the array of substrate binding sites.^33^ Although DnaJB1 generally binds to substrate proteins only transiently, it seems to accumulate on amyloid fibrils and loads several Hsp70 molecules in close vicinity to each other, leading to Hsp70 clustering that results in fibril fragmentation and depolymerization.^57^, ^58^, ^59^

On the other side, class A JDPs have in addition to the βSD1 the ZnF which specifically binds β-sheets, as recently shown for the interaction of DnaJA2 with the p53 core domain.^60^ However, the ZnF apparently does not bind to all types of β-sheets as class A JPDs are not able to target Hsp70 to amyloid fibrils for disaggregation.^57^, ^61^ Additional substrate binding sites might still await discovery.

The non-canonical class B JDPs, like human DnaJB2, DnaJB6, DnaJB7, and DnaJB8, harbor C-terminal of the G/F-rich region a 7-stranded β-sheet, the first half of which is serine-threonine-rich. In DnaJB2, DnaJB6, and DnaJB7 but not DnaJB8, this β-sheet domain is followed by a mostly unstructured tail of 70–110 residues. DnaJB6 and DnaJB8 were shown to efficiently suppress the formation of polyQ aggregation in cells, and the serine/threonine-rich region is essential for this property.^62^, ^63^, ^64^ DnaJB6 is also important for correct biogenesis of the nuclear pore complex in the interphase by suppressing aggregation of FG-nuclearporins.^65^ Thus, this subclass of class B JDPs seems to be more specialized for intrinsically disordered proteins with a high propensity to form amyloid fibrils. Interestingly, ancestral resurrection of the evolution of class B JDPs revealed that the canonical and non-canonical class B JDP appeared prior to proteins with high propensity for amyloid fibril formation like TAU, Fus, APP, IAPP, α-synuclein, prion protein (PrP) and huntingtin,^20^ suggesting that the loss of the ZnF and the gain of a novel C-terminal domain allowed for the evolutionary tolerance towards high amyloid propensity proteins.

More recently, also a class C JDP, DnaJC7, was found to suppress aggregation of amyloid-forming proteins involved in a number of neurodegenerative diseases, including Tau, TDP-43, poly-Q huntingtin exon 1.^66^, ^67^, ^68^, ^69^ DnaJC7 does not feature any similarity to DnaJB6 or DnaJB8 except for the J-domain that is not at its N-terminus as in class B JDPs but close to its C-terminus. AlphaFold structure prediction suggests that DnaJC7 contains C-terminal of its J-domain a segment that resembles a G/F-rich region with a potential autoinhibitory helix (Figure 2e). At the N-terminus, DnaJC7 contains a short intrinsically disordered region followed by three tetratricopeptide repeat (TPR) domains, each consisting of three helix-loop-helix elements of roughly 34 residues (thus the name). TPR domains have been characterized to bind to short peptide sequences, most notably the EEVD-motif at the C-terminus of eukaryotic cytosolic Hsp70s and Hsp90s.^70^, ^71^, ^72^ DnaJC7 was found to recognizes with its TPR2b domain a β-hairpin element in Tau but that low affinity interactions with the other two TPR domains are necessary in addition to prevent Tau aggregation in the presence of seeds. The β-hairpin element is unfolded in the Tau-P301L variant, which is associated with frontotemporal dementia. DnaJC7 had a strongly decreased affinity to the Tau-P301L variant, and a mouse tauopathy model revealed a negative correlation between DnaJC7 abundance and Tau-P301S-1N4R seeds in affected brains.^66^ Furthermore, a genome-wide study associated a mutation in the J-domain of DnaJC7 with amyotrophic lateral sclerosis (ALS)^73^ and the TPR domains of DnaJC7 were found to delay aggregation of the ALS-linked protein TDP-43.^74^ Whether DnaJC7 is also regulated by an autoinhibitory mechanism is unclear at the moment. However, it was recently shown that binding of DnaJC7 to Hsc70 involves an interaction of one of the TPR domains of DnaJC7 with the C-terminal EEVD-motif of Hsc70, and that this interaction is essential for the interaction of its J-domain with Hsc70.^74^

Not all amyloid-interacting JDPs act on preventing amyloid formation or breaking and depolymerizing amyloid fibrils. There is at least one JDP, DnaJC8, that favors the formation of specific amyloids. Amyloids are most commonly considered in the context of pathological states like neurodegeneration. However, there are also physiological amyloids that serve important functions. In several organisms, most notably *Aplysia*, *Drosophila,* and mice, it was shown that cytoplasmic polyadenylation element-binding (CPEB) proteins form prion-like amyloid fibrils during the formation of long-term memory and that this amyloid state is necessary for memory formation.^75^, ^76^, ^77^ Oligomeric but not monomeric Orb2, a CPEB protein in *Drosophila*, was found to interact with the DnaJC8 homolog funes and funes was necessary for the transition of *Drosophila* Orb2 into amyloid fibrils.^78^ Interestingly, *Drosophila* funes had no effect on *Drosophila* Orb2 or on *Aplysia* CPEB when it was purified recombinantly out of *E coli*, suggesting that posttranslational modification play an essential role for its function. Furthermore, funes was very specific for the CPEB proteins and had no effect on the formation of disease-linked amyloid fibrils of TDP-43 or Aβ_42_. For the action of funes the activation of Hsp70’s ATPase is essential, as a funes variant with a deleted HPD motif bound to Orb2 oligomers like wildtype funes, but did not induce the conversion of Orb2 into amyloid fibrils. Transgenic flies expressing funes∆HPD exhibited impaired long-term memory, whereas the short-term memory was not affected. No Orb2 amyloid fibrils were observed in these transgenic flies. Thus, the amyloid-inducing activity of funes together with its interaction with an Hsp70, is essential for the formation of long-term memory.^78^

Apart from amyloid fibril formation, many JDPs have been shown to prevent the aggregation or degradation of proteins at low stoichiometric excess by stabilizing folding intermediates or stress denatured proteins.^79^ This action is not limited to generalist class A or class B JDPs but also found in many class C JDP. For example, the class C JDP DnaJC20/HscB, called Jac1 in yeast, protects its substrate, the Fe-S cluster scaffold protein Isu1, from degradation by the Lon protease Pim1.^80^ In yeast Jac1 interacts with its specialized partner Hsp70 Ssq1 to assist the transfer of the Fe-S cluster from the Isu1 scaffold protein to target enzymes, which is the essential function of yeast mitochondria when growing on fermentable carbon sources. Thereby, Jac1 targets Isu1 to Ssq1 and Ssq1 destabilizes Isu1 to facilitate Fe-S cluster transfer. In *E coli* this task is performed by the Jac1 ortholog Hsc20/HscB, which interacts exclusively with the Hsp70 HscA.^81^ In prokaryotes the *hscB* gene is generally found together with the *hscA* gene (328 of 330 genomes), but the two genes only occur in 19% of the 1700 prokaryotic genomes analyzed.^9^ In the remaining prokaryotic species, other JDP-Hsp70 pairs may take over this task. In contrast, HscB/Jac1 is conserved and essential in all eukaryotic species but not the specialized Hsp70 Ssq1 that only exists in Saccharomycetaceae species. From ascomycota to humans, Ssq1’s function is exerted by the only mitochondrial Hsp70 (Ssc1 in ascomycota; HspA9/mortalin in humans), the Hsp70 that also drives protein import and performs general folding functions in the mitochondrial matrix.^82^ Of note, eukaryotes only inherited HscB from their prokaryotic ancestors. HscA was lost, and Ssq1 evolved independently in a small subgroup of ascomycota.^83^, ^84^ Thus, the JDP is key to this function and can cooperate with generalist and specialist Hsp70s.

Examples for class C JDPs that serve more general functions are the endoplasmic reticulum (ER) JDPs DnaJC10/ERdj5 and DnaJC3/ERdj6/p58^IPK^. ERdj5 contains in addition to the J-domain six thioredoxin domains, four of which are functional, and is the only disulfide reductase in the ER. It stabilizes misfolded proteins, reduces their disulfide bonds, and aids in retro-translocation to the cytosol through the ER-associated degradation pathway (ERAD).^85^, ^86^ DnaJC3/ERdj6 is structurally very similar to DnaJC7/TPR2 in the cytosol (Figure 2e), containing, after the cleavable ER-targeting signal sequence, three TPR domains connected to the J-domain via a short flexible linker and a C-terminal disordered sequence of 42 residues resembling a G/F-rich region but without any indication of an autoinhibitory helix. It cooperates with the ER resident Hsp70 BiP in the refolding of misfolded proteins and, in addition, functions as negative feedback regulator of the PERK arm of the unfolded protein response, ensuring the restart of translation.^87^, ^88^

JDPs are able to remodel their substrate proteins. Interaction of JDPs with substrates can lead to local unfolding, as demonstrated for the interaction of DnaJ with the *E coli* heat shock transcription factor σ^32^ and this unfolding was much more prominent than the DnaK-induced local unfolding.^2^ Interaction of JDPs with protein complexes can lead to disassembly of the complex. The class C JDP Cwc23, one of the few JDPs that are essential in yeast, remodels the spliceosome to disassemble the post-splicing complex. For this activity the J-domain is not essential. In this case Hsp70 might only speed up the disassembling process, accelerating dissociation of Cwc23 from its substrate.^89^, ^90^ Interestingly, overexpression of Cwc23 complements the deletion of the most abundant class A JDP Ydj1 and for this “moonlighting” activity of Cwc23 the J-domain is essential.^90^ Apparently, the distinction between specialists and generalists in JDPs is rather artificial, and many class C JDPs, in addition to their specialized function, might contribute to the general protein quality surveillance function of the generalist JDPs.

## Substrate processing by JDPs bypassing Hsp70s

The Hsp70 chaperone machinery is known to cooperate with Hsp90s in the chaperoning of a distinct class of native proteins, including nuclear receptors, protein kinases, E3 ubiquitin ligases, and many other key regulatory proteins in the cell.^91^, ^92^ The general scheme for this cooperation involves initial interaction of the JDP with the substrate protein, binding of the Hsp70 to the substrate protein, and subsequent transfer to Hsp90 with the help of the scaffolding protein HOP that binds to the C-terminal EEVD motifs of Hsp70 and Hsp90. The successive interaction of the substrate with Hsp70 and Hsp90 is essential for the activation of the substrate. Recently, it was demonstrated that Hsp70 can be bypassed in this reaction with the help of the NudC cochaperone. NudC interacts with the substrate-bound JDP and guides it to the Hsp90 chaperone. This direct transfer of the substrate from the JDP to Hsp90 also leads to efficient activation of the substrate.^93^ It is currently unknown for how many substrates of the Hsp90 system this pathway is an alternative to the participation of Hsp70 or under which conditions such a bypassing of Hsp70 is advantageous.

## JDP with impaired Hsp70 interaction motif

The HPD motif is the most conserved sequence in JDPs, and amino acid replacements like HPN or QPD, as mentioned above, lead to a complete loss of the ability of the JDP to stimulate the ATPase activity of Hsp70. The only replacement tolerated seems to be HPE that is found in the *E coli* DjlB and DjlC, which are known to interact with HscC, an Hsp70 that diverged significantly from the bona fide Hsp70s.^26^ Nevertheless, in sequence databases several JDPs are found with a mutation in the HPD motif, and these presumably nonfunctional JDPs are sometimes called type IV JDPs, although their additional domains would classify them class B or class C JDPs. Whether these deviations from the HPD motif are accidents of evolution or positively selected for remains enigmatic.

For example, the human DnaJB13 is in all features a typical class B JDP, including the N-terminal J-domain, the G/F-rich region with the autoinhibitory helix, the two βSDs, and a C-terminal dimerization domain, closest related to DnaJB1, DnaJB4, and DnaJB5. However, the HPD motif is altered to HPL. DnaJB13 is a component of the radial spoke of the axoneme of primary cilia, and mutations that render it nonfunctional lead to primary cilia dyskinesis and male sterility.^94^, ^95^ According to current knowledge, this JDP should not be able to stimulate the ATPase activity of Hsp70s. Whether it nevertheless interacts with Hsp70s is currently unknown. Interestingly, high-throughput mass spectrometry identified 4 phosphorylation sites within the J-domain of DnaJB13, suggesting that its J-domain is important and subject to regulatory posttranslational modifications (https://www.phosphosite.org).

Another example of a J-domain with mutated HPD motif is Pam16 (DKE in yeast and DKS in human). As mentioned above, it forms a stable complex with the functional J-domain of Pam18 and inhibits the ability of Pam18 to stimulate the ATPase activity of its partner Hsp70 Ssc1.^37^ The mechanistic logics of this association are not clear.

Interestingly, the malaria causing parasite *P falciparum* contains 13 type IV JDPs, 11 of which contain a PEXEL sequence, suggesting that they are exported into the host erythrocyte.^11^ Evidence has been published that some of these exported type IV JDPs are essential for parasite survival^96^ or required for growth or survival during febrile episodes.^97^, ^98^ Whether they interact with Hsp70s remains to be investigated.

## Hijacking the Hsp70 machinery

For many viruses, recruiting Hsp70 is essential for their proliferation. In 137 genomes of bacteriophages, JDP-encoding genes were found, one encoding a class B JDP and 136 encoding class C JDPs, comprising 49 unique sequences.^9^ Of these 48 class C JDPs had no other known domain next to the J-domain. One of these JDPs, Rki of the T4-related bacteriophages RB16, RB42 and RB43, was shown to interact with DnaK to release and stabilize the heat shock transcription factor σ^32^ to induce the heat shock response, resulting in the increase of the levels of chaperones presumably to cover their protein folding needs.^99^

JDPs are also found in mammalian polyomaviruses.^22^ The best-investigated viral JDP is the T-antigen of SV40.^100^, ^101^, ^102^, ^103^ The functional J-domain of the T-antigen is essential for its function in DNA replication, proliferation, and cellular transformation. A BLAST search revealed that the J-domain is encoded in the genomes of many, if not all, polyomaviruses of a wide variety of mammalian species.

Other viruses seem to encode functional analogs to JDPs. Human Herpes simplex virus-1 immediate early protein ICP22 has distant sequence similarity to the J-domain of DnaJB1, but no HPD motif, and to the βSDs. ICP22 pulls down Hsc70 in an ATP-dependent manner like many JDPs and recruits Hsc70 to specific loci in the nucleus of infected cells full-filling the targeting function like JDPs.^104^ ICP22 is well conserved within the Herpes simplex viruses. The absence of an HPD motif might be considered to exclude the function as a J-domain. However, a recent study with de-novo-designed analogs of J-domains produced a sequence that stimulated the ATPase activity of Hsc70 and could assist refolding of denatured luciferase when grafted onto *E coli* DnaJ, replacing the native J-domain.^105^ This sequence lacked the HPD motif but had properties of a functional J-domain.

Not only viruses use JDPs to exploit the Hsp70 machinery. The intracellular pathogen *P falciparum* exports 17 JDP into the hosting erythrocyte, and at least some of them are able to functionally interact with the human Hsp70s.^106^, ^107^

## Conclusions

**Fig. 3:**
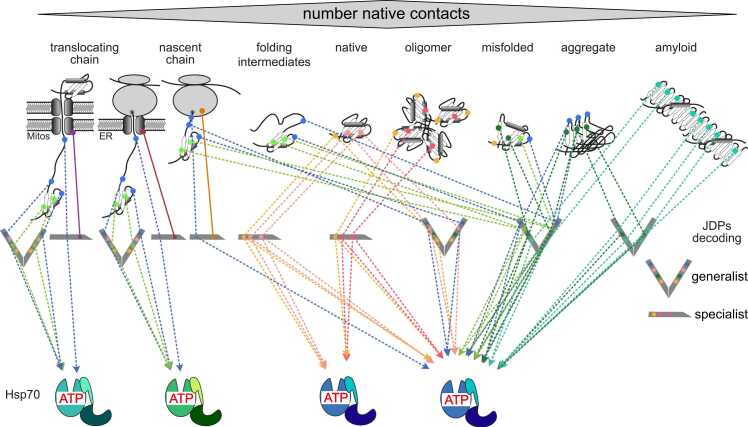
*Model for the JDP network logic.* A selection of protein folding processes involving the Hsp70 network. On their folding trajectories, proteins transit through different conformations, exposing hydrophobic residues that are found in the native state in the hydrophobic core. Even native proteins might expose linear or tridimensional motifs recognized by JDPs, in particular in their monomeric state before assembling into oligomeric complexes. Stress denaturation, aggregation, and amyloid fibril formation again lead to exposure of motifs that distinguish them from their native folds. These complex patterns of hydrophobic, hydrophilic, and charged residues, illustrated as colored dots, are decoded by the multiple low-affinity substrate-binding sites of JDPs that recognize in this way whether a protein is in its native state or unfolded, folding intermediate, misfolded, non-native oligomer, aggregate, or amyloid and in need of an Hsp70 and then flag the substrate proteins for the different Hsp70s found in the respective cellular compartment. Generalist JDPs as class A and canonical class B JDPs are illustrated as dimeric V-shapes; non-canonical class B’ and class C JDPs are illustrated as linear arrangements of substrate binding sites, independent of whether or not they form oligomeric assemblies. JDPs that are tethered to the ribosome or translocation pores are linked by solid lines to their respective location of action. Dashed lines indicate recognition/decoding of folding features by JDPs and targeting to Hsp70s.

The picture emerges that class A, class B, and some class C JDPs present several low-affinity binding sites in a defined geometry and use combinatorics to scan the surface of proteins for features that are characteristic for the unfolded, misfolded, denatured, pathological oligomeric, aggregated, or amyloidic state (Figure 3). Such combinatoric interactions are reminiscent of logical gates. How the decoding of the non-native state works in detail, in particular how rigid or dynamic the geometry of the recognizing low-affinity binding sites can be remains to be elucidated. It is also currently unknown whether all J-domains that interact with the same Hsp70s stimulate each of the Hsp70 with similar efficiency or whether there are hierarchies of interaction that allows for prioritization of specific substrates. Of special interest for future studies is also whether JDPs with non-conservatively replaced HPD motif rewire the JDP network by competing with functional J-domains for interaction with Hsp70s, thereby inhibiting a potential unfolding action of the Hsp70 chaperone, or whether they serve as recruiters of Hsp70s to enrich their concentration at specific cellular locations without stimulating their ATPase activity and thus do not convert them into the low-affinity state.

The more we understand the logics of this network, the better this knowledge could be harnessed for screening for specific inhibitors or activators that might be developed into drugs for therapeutic purposes. Small molecules that inhibit the JDP-stimulated ATPase activity but not the intrinsic ATPase activity of Hsp70s already exist.^108^ Moreover, a small molecule was discovered that bound close to the J-domain interaction site on Hsp70 and activated Hsp70 in the absence of and in synergy with JDPs.^109^ Introduction of a bulky group converted this activator into an inhibitor of the J-domain-Hsp70 interaction. The challenge will be to develop small molecules that are specific for individual JDP-Hsp70 pairs. Such high-precision tools will be more suitable for targeted treatment of specific diseases that involve Hsp70-JDP network components such as malignant transformation, neurodegeneration, inflammatory diseases, autoimmunity, and infections with viral, bacterial, fungal, or protozoan parasites.

Pioneering researchers like Len Neckers, who were the first to discover that chaperones can be inhibited, thereby curbing tumor growth,^110^, ^111^ and who, early on, took on the challenge of translating insights into protein folding and chaperone networks into drug discovery programs for the benefit of patients, continue to inspire chemical biologists today to pursue this path.

## CRediT authorship contribution statement

**Matthias P. Mayer:** Writing – review & editing, Writing – original draft, Visualization, Funding acquisition, Conceptualization.

## Declaration of Generative AI and AI-Assisted Technologies in the Writing Process

No generative AI has been used during the writing process.

## Declarations of interest

The authors declare that they have no known competing financial interests or personal relationships that could have appeared to influence the work reported in this paper.

## Data Availability

No data was used for the research described in the article.
